# Social, Environmental and Economic Impact Assessment of COVID-19 on Rural Tourism

**DOI:** 10.3389/fpubh.2022.883277

**Published:** 2022-05-10

**Authors:** Fatemeh Eslami, Razieh Namdar

**Affiliations:** Department of Agricultural Extension and Education, College of Agriculture, Shiraz University, Shiraz, Iran

**Keywords:** sustainable rural tourism, economic effects, social effects, environmental effects, COVID-19

## Abstract

Today, various solutions have been proposed to improve the economic situation of villages and deprived areas, among which tourism is known as the best solution for those areas with the necessary potentials for tourism development. On other hand, the COVID-19 pandemic had significant effects on human life worldwide. The prevalence of COVID-19 has caused a lot of damage to different sectors of the global economy, but without a doubt, the rural tourism industry should be considered among the economic activities that have suffered the most from this virus. In this study, with the aim of investigating these effects on the rural tourism industry, it has been analyzed and compared in three important economic, social and environmental dimensions before and after the outbreak of the COVID-19. This quantitative study was used survey method. The statistical population of the study consisted of local stakeholders of rural tourism and experts of the relevant organizations in Natanz county of Iran. The results of confirmatory factor analysis indicate that the constructs used in the model have appropriate and acceptable fit. The results of the study also, showed that the prevalence of COVID-19 has adverse consequences including reducing the desirable economic and social effects of tourism mentioned among both groups of experts and rural stakeholders. from rural tourism stakeholders' opinion, environmental variables of the tourism areas before and after the COVID-19 was different, and in the absence of tourists in this area, the destructive environmental effects have strongly decreased.

## Introduction

Todays, it is not sufficient speak only about agriculture to meet the needs of rural communities. The agricultural sector cannot be assessed on the basis of production, income and employment; since, in addition to production, agriculture should also be evaluated on the basis of some other concepts such as protection, recreational activities and leisure, non-consumption values, etc (1). In fact, it is necessary to use the innovation variable to find new options for livelihood in traditional rural and agricultural areas (2). Tourism is considered as the set of phenomena and connections resulting from the interaction between tourists, capital, host governments and communities, universities and non-governmental organizations in the process of attracting, transporting, receiving and controlling tourists and other visitors (3).

According to Brandt land report in 1987, for development to become a sustainable paradigm (4), tourism development can be considered sustainable just when it can continue indefinitely in an environment, no harms suffer humans and environment, and also does not harm the development of other social activities and processes (5).

Nowadays, one the tools of sustainable growth and development is tourism, which is increasingly spreading around the world. At present, many countries that are concerned about their economic and social development consider the tourism industry as an important and fundamental necessity. Placing this category in the household basket calls for increasing communities' awareness of sustainability, the limitations resulting from the overuse of resources and energy, communities' attitudes about the environment and its relationship with tourism (6). Tourism must be developed not only with the requirements of tourism development, but also in accordance with the natural environment. Hence, the positive effects of this industry should also be revealed on the environment. It is important to strengthen the scientific concept of ecological tourism, to recognize the concepts of sustainable development, to respect for tourism and environmental resources in the whole society, and to develop propaganda on the value and importance of tourism resources (7). Tourism has changed dramatically over the last 40 years (8, 9) and is clearly recognized as an independent scientific category.

Tourism for decades, around the world has grown rapidly. Tourism is a major driver of growth in many countries and regions (10). The tourism industry is one of the fastest growing industries in the world economy and enjoys government support in various countries (11). This is of great importance so that the tourism industry is the fourth top industry in the world after the automotive, construction and food industries (12). Tourism is an important export sector and can act as a driver of economic growth (13). This is why policymakers and planners in any country need careful planning to increase revenue through tourism policies, however, this should not jeopardize sustainable tourism development (14). Various studies conducted in different parts of the world show that along with rapid growth, the negative effects of tourism also have been spreading.

Due to the continuous development of the tourism industry, its harmful effects become more severe and more diverse from year to year (15). Therefore, it is important to study and identify the effects and consequences of tourism activities.

## Literature Review

Rural tourism has long been considered a potential tool for socio-economic development and rural revitalization. The development of rural tourism has become a common policy in developed and developing countries (16).

The effects of tourism development in various dimensions, including negative socio-cultural effects, have been considered and emphasized in literature. Economic effects of tourism (17) include items such as: unequal distribution of income, rising prices and poverty (18), increased corruption, reduced capacity of residents to meet living needs, and increased environmental costs (19), harm to natural heritage and ecological-cultural Security (20), negative impacts on environment, society, culture and even economy (21), improving participation and learning opportunities between people (22), and finally capital and cultural security (19).

One of choices with significant tourist attractions, especially the traditional lifestyles and cultural values, are the surrounding rural areas, which have been able to meet the expectations and demands of postmodern tourists and has made the development of rural tourism a common policy in developed and developing countries (16).

Given the decline of agricultural production systems in the last decade, the importance of preserving, maintaining and managing indigenous, ancient and ecological sites has attracted global attention, since the preservation of these areas has considerable potential to contribute to sustainable livelihoods, to attract tourists and to conduct scientific research (23). Ecological tourists can be both supporters and motivators of ecotourism resources (7). Tourism phenomenon can have different positive and negative effects on the rural environment. In other words, studies have shown that how rural tourism can be used as an effective tool for economic growth when agriculture cannot be the only source of livelihood (24).

The success of the tourism industry depends on recognizing, understanding, and the support quality of local residents and hosts. Therefore, understanding the reaction of the local community and the host to the effects of tourism is essential to achieve the ideal support of rural communities for sustainable tourism development (25). Recognizing the effects of tourism to achieve to sustainable development will not be possible without the involvement of stakeholders (26).

The research background on the effects of tourism in rural areas dates back to the late 1960s and early 1970s (27, 28). The experiences of research in the past decades show that researchers divided the effects of tourism into positive and negative parts and studied them in economic, social and environmental dimensions (29, 30). In general, the effects of rural tourism can be defined as the results of a relatively complex process between tourists, hosts, and host settlements (31).

At present, the emerging disease of COVID-19 has been able to affect many popular activities. Since late 2019, Coronavirus epidemic (COVID-19) has had unprecedented and profound negative effects on global society and economy. The disease has been declared a global threat by the World Health Organization (WHO) which propagated rapidly in 205 countries, almost all around the world (32).

The spread of Covid was increasing until March 11, when the President of the World Health Organization declared COVID-19a pandemic disease and the world entered a new phase of the disease (33). As the new dimensions of the disease was distinguished, governments started new policies, including quarantine of infected cities, staying at home, social isolation, banning communities, and closing educational institutions. These led to slowing the socioeconomics trend of communities' life (34).

COVID-19 has had a significant impact on human life around the world. The WHO's report in May 10, 2021 shows that the Corona has resulted in the death of almost 5.9 million people and more than 427 million confirmed cases worldwide (35). The United Nations World Tourism Organization (UNWTO) reported that since April 20, 2020, all major tourism destinations have imposed travel restrictions in response to the Corona epidemic. Tourism is one of the industries that is negatively affected by this epidemic. Lockdowns in many countries, widespread travel restrictions, and the closure of airports and national borders reduced the number of international tourists arriving in the first quarter of 2020 to 67 million. This decrease means a loss of approximately $ 80 billion in tourism revenue, compared to the same period in 2019 (36). COVID-19 has became a complex and pervasive disease that humanity is suffering from. This epidemic has had a profound effect on social and economic systems, health and development. It also has had socio-psychological effects on individuals, families, social groups, companies and nations all around the world (37–39).

In a study examining the threat of the Corona virus and real-time impact on the tourism threat, Baum and Hai (2020) showed that the outbreak of the Corona virus had a significant impact on the tourism industry and a 100 percent reduction in the industry's revenue in some regions of Asia, Europe and North America.

In another study, at the time of the Corona, Wan et al. (7) Examined the negative effects of the Corona virus on the tourism industry and changes in the lifestyle of tourists and the behaviors and preferences of travelers. The results showed that the COVID-19 virus crisis was affecting travel and tourism patterns, and that in the future, tourism industry activities will be based on smart tourism, and that these changes will force businesses in the industry to reconsider their service design to survive.

Tourism is a commercial industry that has suffered a lot from the COVID virus. The outbreak of COVID-19 has caused a great deal of damage to various sectors of the global economy. But without a doubt, the tourism industry should be considered as one of the economic activities that has suffered the most from the virus. In this study, these effects on the tourism industry have been studied and this has been analyzed and compared in three important economic, social and environmental dimensions before and after the outbreak of COVID-19 virus. The results of this study provide suitable suggestions for managers and tourism experts of the city as well as many decision makers in this field to plan to reduce the negative effects of rural tourism.

## Methodology

### Research Type

The data needed to analyze the research questions were collected using a questionnaire. The questionnaire was extracted using research hypotheses as well as previous articles evaluating the effects of tourism. The questionnaire has three categories of questions in the field of economic, social, and environmental variables. To assess the reliability of the questionnaire, Cronbach's alpha method was used using SPSS software version 26. Its face validity was confirmed by the professors of the Department of Agricultural Extension and Education in Shiraz University. In order to answer these questions, 5-Point Likert Scale have been used.

The aim of this study was to evaluate the social effects of COVID-19 pandemic on sustainable rural tourism in Natanz city. Data analysis of this research was performed in two parts: descriptive and analytical or inferential statistics. In this study, a questionnaire was used and SPSS26 and LISREL software were used to analyze the data.

The study area in this study was the tourism hub of Natanz city, which shines like a green jewel between the dry and desert cities around it and is one of the most important historical and tourist cities in Iran and attracts many tourists annually. Natanz county is subdivided into two districts: the Central District and Emamzadeh District. This county is consisted of four cities: Natanz, Badrud, Khaledabad & Tarq. The most important tourist villages from both parts including Abyaneh (one national works of Iran), Barzroud and Hanjan, Toroghroud and Kesheh and Matinabad were selected for sampling.The city of Natanz, with 1,800 historical monuments throughout the year, has been located on the main North-South main route of Iran, and has welcomed many travelers, tourists and Orientalists throughout Iran's history. In 2017, this figure reached 47,500 tourists from foreign countries (40).

### Sample Size and Statistical Population

Sampling was done from the two central parts and Imamzadeh Agha Ali Abbas of this city (Abyaneh, Borzroud and Hanjan villages, Toroghroud, Kesheh and Matin Abad). The villages of Abyaneh—which is registered as one of the national monuments of Iran—are Torghroud, Kesheh, Hanjan, Borzroud and Matinabad (41). The statistical population of the study included two groups of villagers, tourism stakeholders, residents of tourism target villages in one hand, and experts and providers of tourism services in Natanz city in other hand.

The present study is a kind of quantitative applied research in which the cross-sectional survey method has been used. Two methods of library study and face-to-face interview with participants were used to collect data. Random sampling method was used to select the samples. Cochran's formula was used to determine the sample size required for the present study. According to the latest census of the Statistics Center of Iran in 2016, the number of households in the tourist villages of Natanz was about 610, which was obtained using the Cochran's formula and taking into account the error rate of 0.06, the sample size was estimated 235. Equation 1 shows the values of each of the Cochran's formula parameters and the sample size determination process.


(1) $$\begin{array}{r} n=\frac{Nz^{2}pq}{Nd^{2}+z^{2}pq} \end{array}$$

Also, the number of experts in different departments (roads and buildings, Red Crescent, municipality, private companies, agricultural jihad, natural resources, cultural heritage, governorate) based on Cochran's formula, 110 samples were obtained, which is also based on the number of experts in each department was distributed and completed.

### Research Limitation

In this study, restrictions on the spread of the corona virus, access to tourists, locals and data collection, as well as travel to tourist areas faced difficulties.

## Research Analysis and Results

Participants in the study in terms of gender included 38 women (34.5%) and 72 men (65.5%). The average age was 42 years and their age range was 28 to 55 years. In terms of education, most of them had bachelor degree.

The frequency distribution of local stakeholders based on gender showed that 68 (28.9%) of the participants in this study were rural women and 167 were male (71.1%). The average age of local stakeholders was 45 and their age range was between 10 and 76 years. The average level of education of local stakeholders was 9 years. The highest frequency of jobs was related to agriculture and animal husbandry with 82 people. The highest frequency of income included people working in the tourism industry (30%).

### Confirmatory Factor Analysis Results

To evaluate the social effects of COVID-19on rural tourism better and more accurately, structural equations and confirmatory factor analysis were used with LISREL 8.8 software. After evaluating the correlation coefficients between the variables used in the research, based on the answers provided by the villagers or local stakeholders, the data entered the confirmatory factor analysis, since this sample group had statistical logic in terms of number and ratio of respondents to questionnaire items. Obvious variables were entered into confirmatory factor analysis to measure the economic effects of the research. The standardized factor load of the indicators in t-factor analysis and their significance level with respect to the first order value are given in Table 1, According to the obtained results, the amount of factor load of the structures of economic effects is >0.3 and the value of t is >1.96, so all the structures used in this dimension are approved due to the acceptable reliability in the model. Based on the obtained coefficients of factor loads, some of the most important economic effects can be mentioned as follows: increase in employment in the tourism field with a standard coefficient (0.94), the employment of women in tourism occupations (with a standard coefficient of 0.89), Local community satisfaction with tourism revenue (with a standard coefficient of 0.95), improving the level of wages in the touristic area (with a standard coefficient of 0.95).

**Table 1:** Summary of results obtained from the economic effects model (confirmatory factor analysis).

**Structure**	**Item**	**Indicator**	**Standard coefficient**	**Standard error**	**T Value**
Economic	Increase of job creation in the touristic area	EA1	0.94	0.1	9.77
	Employment status of women in touristic jobs	EA2	0.89	0.1	10.78
	Local community satisfaction with tourism revenue	EA3	0.95	0.12	10.19
	Purchasing power of people working in tourism	EA4	0.72	0.09	8.12
	Increase in unemployment of people active in tourism activities in the region	EA5	0.78	0.07	10.79
	Reduction of seasonal and permanent unemployment rates in the touristic area	EA6	0.62	0.06	10.14
	Creation of small job opportunities for the residents of the touristic area	EA7	0.63	0.06	10.73
	Creating job opportunities in the touristic area	EA8	0.78	0.17	10.79
	Improvement in the level of wages in the touristic area	EA9	0.95	0.16	8.37
	Activities of small and medium local investors in the region	EA10	0.76	0.11	9.86
	Status of bank facilities and loans for tourism	EA11	0.9	0.08	10.81
	Establishment of small and medium local economic enterprises in the region	EA12	0.38	0.04	10.65
	Tax increase due to local government spending in the region	EA13	0.71	0.07	10.54
	Investment in touristic villages	EA14	0.44	0.04	10.8

Also, according to the results mentioned in Table 2, the amount of factor load of social impact structures is >0.3 and the value of t is >1.96, so all the structures used in this dimension remain in the model due to their acceptable reliability. Based on the standard coefficients, these items have a higher level: the level of residents' trust in government agencies (standard coefficient: 0.96), the level of residents' trust in private institutions and companies (standard coefficient: 0.94), the status of tourists' assistance to the poor villages (standard coefficient: 0.85), interest in living with people of other religions or denominations (standard coefficient: 0.95), disappearance of indigenous culture in the region (standard coefficient: 0.96).

**Table 2:** Summary of results obtaine+d from the social effects model (second-order confirmatory factor analysis).

**Structure**	**Item**	**Indicator**	**Standard coefficient**	**Standard error**	**T** **Value**
Social	The level of residents' trust in government agencies	SA1	0.96	0.19	10.82
	The level of residents' trust in private institutes and companies	SA2	0.94	0.16	10.82
	The level of residents' trust in the local council and the local village administration	SA3	0.89	0.16	10.82
	Competition among the villagers in attracting tourists	SA4	0.85	0.1	10.81
	The situation of tourists helping the poor villagers	SA5	0.9	0.08	10.81
	Status of holding traditional ceremonies and celebrations	SA6	0.47	0.04	10.81
	The mood and vitality of the villagers	SA7	0.28	0.03	9.77
	Status of construction activities, including roads and welfare centers	SA8	0.75	0.07	10.82
	Preservation of traditional buildings in the touristic area	SA9	0.45	0.04	10.81
	Cultural pride and confidence of the locals	SA10	0.32	0.03	10.81
	Disturbance situation with the arrival of tourists for the villagers	SA11	0.8	0.07	10.82
	Motivation for more literacy among villagers to communicate with tourists	SA12	0.46	0.04	10.78
	Trust to neighbors and friends for help when needed	SA13	0.87	0.09	10.82
	Compromission of local people in times of conflict	SA14	0.53	0.05	10.79
	Interest in meeting and living with people with different dialects and languages	SA15	0.84	0.08	10.82
	Participation in activities and tasks that are not within the scope of duties	SA16	0.48	0.04	10.82
	Interest in living with people of other religions or denominations	SA17	0.95	0.18	10.82
	Voluntary participation in the construction of public buildings in the area	SA18	0.54	0.05	10.82
	Participation in programs for the development of the region	SA19	0.53	0.05	10.79
	Participation to strengthen charity organizations and foundations	SA20	0.54	0.05	10.82
	Help to solve problems for locals	SA21	0.54	0.05	10.81
	Satisfaction of the people of the region with the level of support for the expansion of cultural and local activities	SA22	0.81	0.07	10.82
	Welfare of the villagers due to the construction of roads and public facilities	SA23	0.52	0.05	10.82
	Preservation of cultural and historical values and patterns of the region	SA24	0.53	0.05	10.79
	Weakening of indigenous culture in the region	SA25	0.96	0.18	10.82
	preservation traditional customs and rituals in the region	SA26	0.81	0.07	10.82
	Lack of maintenance and restoration of archeological and historical monuments in the region with the presence of tourists	SA27	0.28	0.03	9.77
	Change in the clothing type of local villagers with the presence of tourists	SA28	0.28	0.03	9.77
	Change in the language and dialect of the local people with the presence of tourists	SA29	0.28	0.03	9.77

The results of the study showed that factor load amount of the environmental impact structures is >0.3 and the value of t is >1.96, so all structures used in this dimension are approved and remain in the model due to their acceptable reliability. According to Table 3, the quality of hygienic affairs in the touristic area (standard coefficient: 0.95), the pristine nature of touristic villages (standard coefficient: 0.95), the preservation of archeological and historical monuments in touristic areas (standard coefficient: 0.55), more beautiful landscape of touristic villages (standard coefficient: 0.56), observance of environmental cleanliness in touristic areas (standard coefficient: 0.54) have a higher order.

**Table 3:** Summary of results obtained from the environmental impact model (second-order confirmatory factor analysis).

**Structure**	**Item**	**Indicator**	**Standard coefficient**	**Standard error**	**T** **Value**
Environmental	Appropriate medical services in the area and the health of local stakeholders	EnvA1	0.38	0.04	10.81
	Quality of hygiene in the touristic area	EnvA2	0.95	0.18	10.82
	Sanitary disposal of waste in touristic villages	EnvA3	0.53	0.05	10.79
	Extinction of animal species and activities such as hunting in touristic villages	EnvA4	0.28	0.03	9.86
	Degradation of plant species in touristic villages	EnvA5	0.28	0.03	9.86
	The amount of manipulation in the natural environment to attract tourists	EnvA6	0.34	0.03	7.87
	The effects of tourism on destruction of natural resources	EnvA7	0.28	0.03	9.86
	Damage to orchards around touristic villages	EnvA8	0.28	0.03	9.86
	Traffic situation in touristic villages	EnvA9	0.31	0.03	9.91
	Noise pollution	EnvA10	0.32	0.03	9.92
	The naturalness and virgin pristine nature of touristic villages	EnvA11	0.95	0.13	10.82
	Maintaining land use in the touristic areas	EnvA12	0.53	0.05	10.79
	Construction of roads and make life easier for local stakeholders	EnvA13	0.44	0.04	10.82
	Increase in beautifulness the landscape of touristic villages	EnvA14	0.56	0.05	10.82
	Observance of capacity threshold and environmental tolerance in the touristic areas	EnvA15	0.51	0.05	10.78
	Observance of the environment cleanliness in touristic areas	EnvA16	0.54	0.05	10.79
	Preservation of ancient and historical monuments in touristic areas	EnvA17	0.55	0.05	10.79
	Preservation and protection of ecosystems and national parks	EnvA18	0.54	0.05	10.79

**Figure 1:**
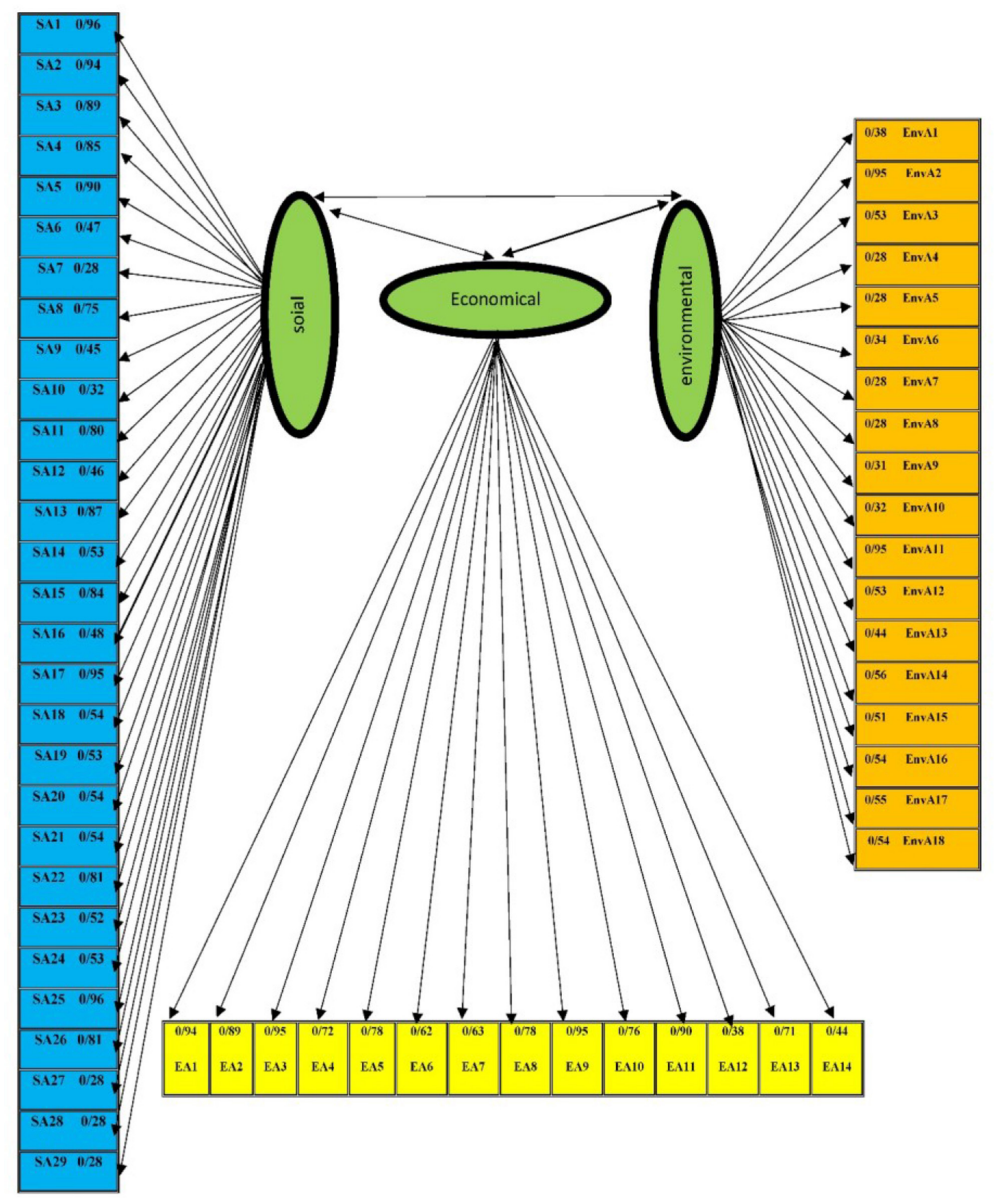
Estimate Model. Chi-square = 3612.23; df = 1775; P–value = 0.0000; RMSEA= 0.067.

The evaluation of the proposed indicators approved that in general, the proposed structural equations model is a suitable model (Table 4). A summary of fit indices of the confirmatory factor analysis model is presented in Figure 1.

**Table 4:** Standard values and fit indicators of social impact assessment model.

**Indicator**	**Standard level**	**Fitted model values**
Chi Square / Degree of Freedom(*X^2^/df*)	3≥	2.03
Normed Fit Index (NFI)	90≤	0.94
Non-Normed Fit Index (NNFI)	90≤	0.96
Comparative Fit Index (CFI)	90≤	0.91
Goodness of Fit Index (GFI)	90≤	0.86
Adjusted Goodness of Fit Index (AGFI)	≤ 90	0.94
Increasing fitness index (IFI)	90≤	0.95
Root mean square residual (RMR)	0.05≥	0.065
Root mean square error of approximation (RMSEA)	0.08≥	0.067

### Comparison of the Average Opinions of Experts and Local Stakeholders in Three Dimensions of Social Effects

**Figure 2:**
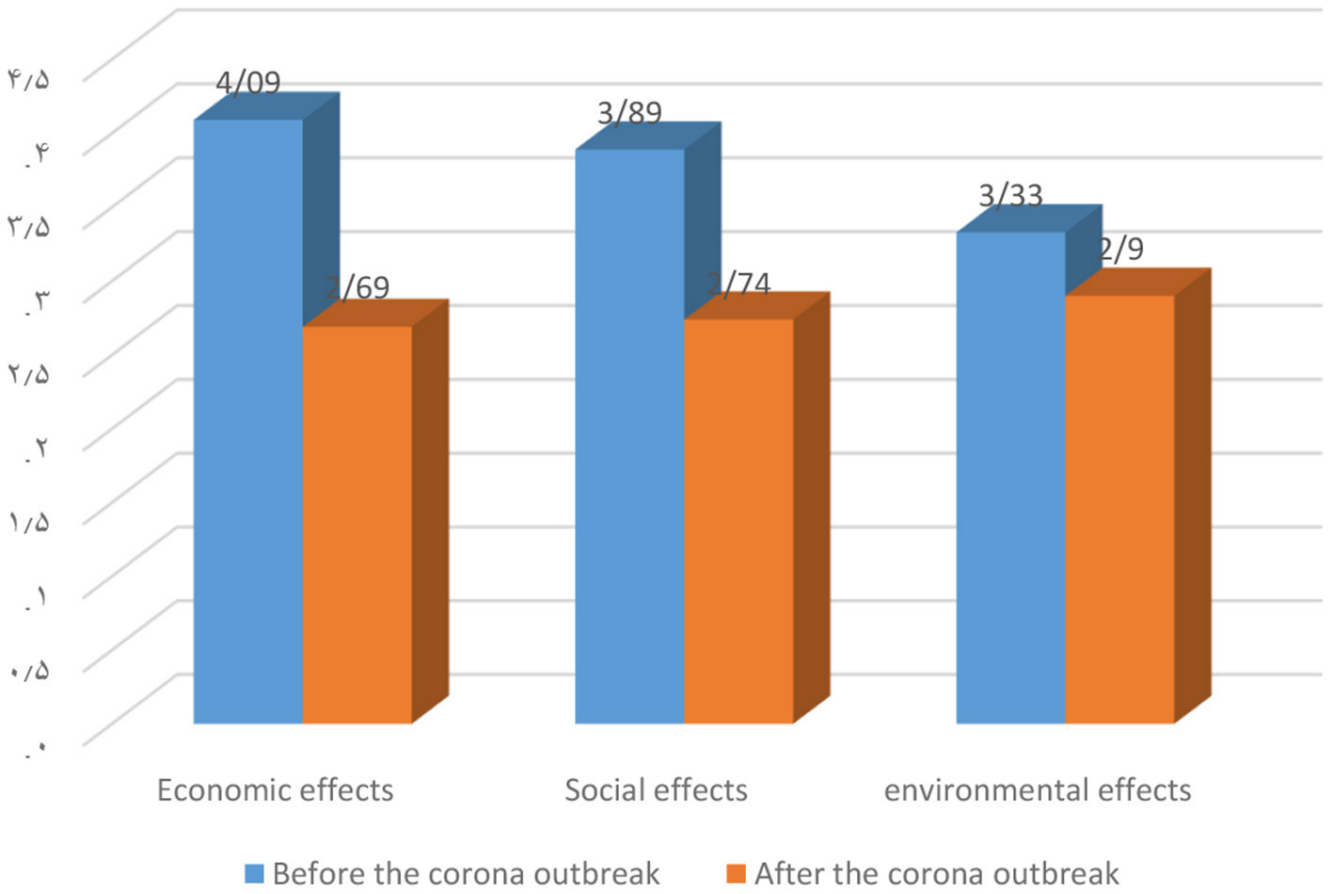
Assessing the economic, social and environmental impacts of the Corona outbreak in experts' opinion.

Using statistical techniques, the opinions of experts and villagers were examined in terms of impact assessment in three dimensions of economic, social and environmental dimensions. Figure 2 shows the comparison of the average responses of tourism effects before the Corona outbreak and after that. Accordingly, the economic, social and environmental effects of tourism in the region have decreased after the outbreak of the virus. In other words, from the perspective of experts, the prevalence of this virus has caused negative effects in all three dimensions in the region.

**Figure 3:**
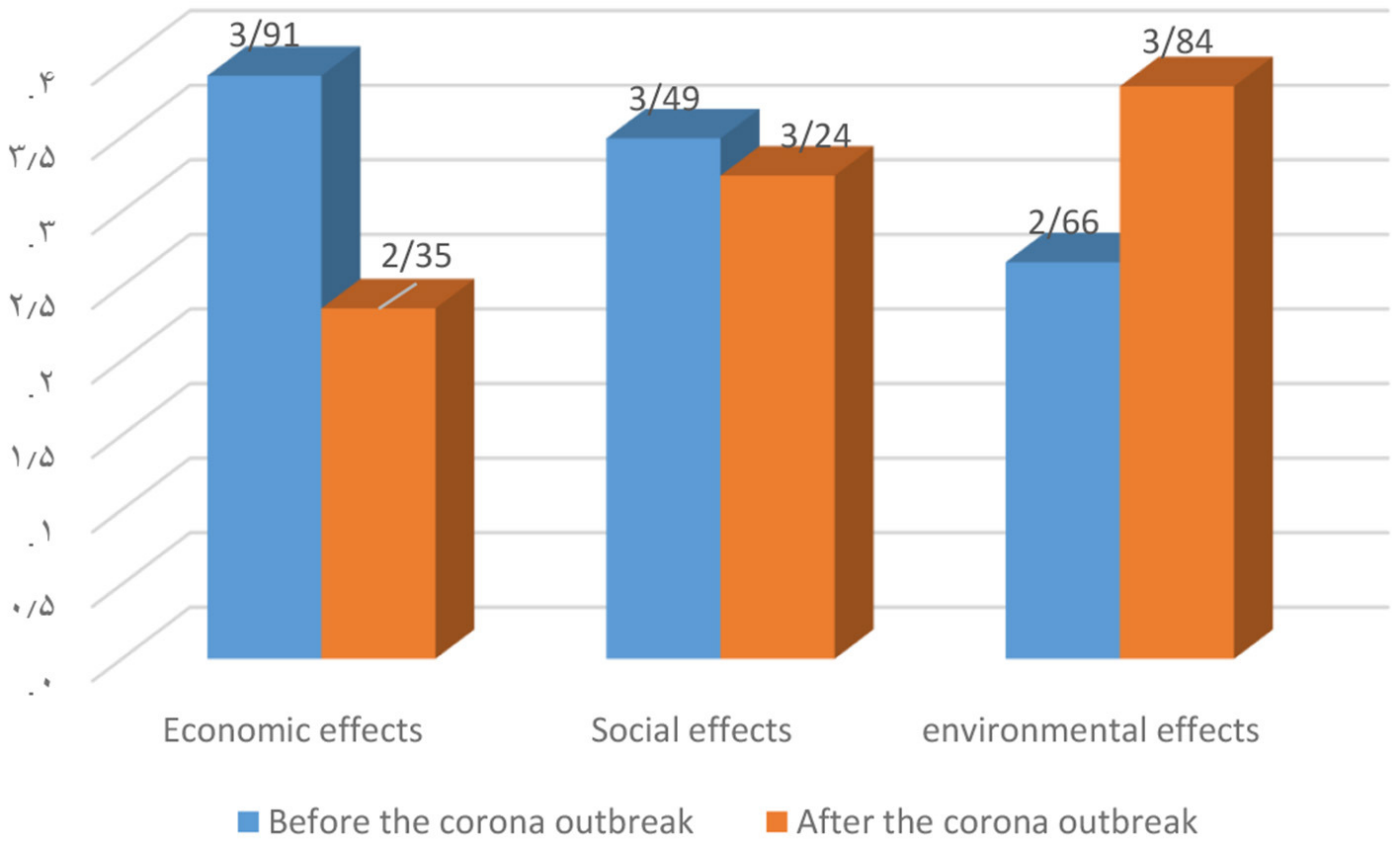
Economic, social and environmental impact assessment chart, before and after the CORONA outbreak from the perspective of local stakeholders.

Figure 3 shows the average economic and social impact of tourism before and after the Corona outbreak on the views of local stakeholders. In both dimensions of economic and social effects, the tourism situation is more favorable for local people, and Corona has caused negative effects on these two variables in the region. Based on differences between average delivered in Figure 3, three aspect including economic, social and environmental effects significantly different before and after the coronavirus outbreak. But in the environmental dimension, this is completely different. Local stakeholders believe the Corona outbreak has reduced the negative environmental impact of tourism in the region. There is a disagreement between experts and local stakeholders in the environmental dimension. Apparently, the environmental negative impacts of tourism in the region include some issues such as: the quality of medical services, health and hygiene, collection and disposal of the wastes, the preservation of ecosystems, national parks and protected areas, preservation of archeological and historical monuments of the region, the extent of destruction of the natural space of the village and reduction of environmental pollution by rural people has been more evident.

Comparing these two above-mentioned graphs, it can be concluded that the difference in economic effects before and after the outbreak of the virus among local stakeholders' opinion is greater than experts. Also, the difference in social effects before and after the outbreak of this virus among experts is more than local stakeholders. Perhaps this disagreement can be due to the fact that local stakeholders in the rural environments are more associated with the environment, tourists and the tourism industry and have a more accurate and complete understanding of the environment, and in contrast, experts have considered the economic and revenue aspects more than other ones.

## Conclusion

The results of confirmatory factor analysis tests show that the obvious variables used in the model to measure the three dimensions of social impact of tourism as a result of Coronavirus outbreak have desirable and appropriate standard coefficients. Therefore, based on the study of good fit indices, it can be concluded that the structures used in the model have a suitable and acceptable fit. It can also be concluded that in general, the proposed confirmatory factor analysis model is a suitable model and the social impact assessment index can be measured correctly in all three dimensions. Also, based on the general and specific objectives of this research, the following important results can be inferred:

### Comparison of Average Economic Effects Before and After Coronavirus Outbreak Between Experts and Villagers

Comparison of the average economic and social effects of tourism before and after the outbreak of Corona virus showed that the views of experts and villagers are in line with each other. Both groups of respondents agreed on the negative economic effects of tourism after the Corona outbreak. But these negative effects are more tangible in experts' responses and it seem that they perceive the current bad economic situation more than villagers. These effects include the loss of employment among rural men and women, the diminishing role of women in monetization, the decline in rural incomes, and so on. For this reason, the need to pay attention to planning and compensatory measures on the employment situation and the economy of tourism stakeholders is becoming more apparent. One of the measures that can be taken during the Corona outbreak to improve the economic situation of the villagers is to develop alternative and complementary jobs. For example, managers and planners can provide opportunities for the development of virtual activities to the villagers or to facilitate a way for the sale of villagers' products through cyberspace.

### Comparison of Mean Social Effects Before and After Coronavirus Outbreak Between Experts and Villagers

Both groups participating in the study had similar views on the negative effects of Corona on social factors of tourism and believed that the Corona virus has reduced the positive effects of social relations, including reduced participation and solidarity among rural people. Under normal circumstances and before the outbreak of the virus, according to experts and local stakeholders, the arrival of tourists have had short-term and long-term negative effects on the sociocultural dimension of rural communities. They have been able to cause negative social effects such as the destruction of cultural customs and traditions. The prevalence of Coronavirus and the decrease in tourist arrivals have been able to reduce these effects. It is suggested that for healing this problem in touristic areas, some strategies could be run, such as holding training courses for how to deal and associate with tourists, as well as culturalization to preserve traditional customs. On the other hand, the outbreak of Coronavirus has caused villagers to be trapped in their homes, which can be solved by holding programs in villages such as rural and seasonal festivals, public sports or cultural and recreational events in compliance with the entire hygienic guidelines. This can prevent the demoralization of the villagers.

### Comparing the Mean of Environmental Variables Before and After the Outbreak of Coronavirus From the Perspective of Experts and Villagers

The views of the two groups participating in the study were not similar on environmental impacts. The overall average of environmental effects from the perspective of villagers before Corona has shown a much lower number than experts, and this indicates that the villagers in general were dissatisfied with the environmental impact of rural tourism, especially about the quality of hygienic and health services, the destruction of the village, the pollution of the environment, the crowds and the excessive traffic of tourists. It can be concluded that with the prevalence of Corona and the lack of tourists entering the area, the destructive environmental effects have been minimized. The amount of traffic has decreased and the crowds and pollution caused by the arrival of tourists have also decreased. But according to experts, on average, these effects are less discussed. In order to prevent the negative environmental effects of tourism in rural logic, it is possible to limit and control the number of tourists by careful planning according to the capacity of the village. It is possible to diminish environmental degradation by using educational brochures in rural areas as a reminder for tourists, as well as using indigenous rural people as rangers, and preserving natural resources and pristine rural environment. It is beneficial to help the protection of valuable touristic areas, historical buildings and the village landscape by using specialized people.

## Data Availability Statement

The raw data supporting the conclusions of this article will be made available by the authors, without undue reservation.

## Ethics Statement

Ethical review and approval was not required for the study on human participants in accordance with the local legislation and institutional requirements. The patients/participants provided their written informed consent to participate in this study.

## Author Contributions

FE: data gathering and literature review. RN: supervisor and research design. Both authors contributed to the article and approved the submitted version.

## Conflict of Interest

The authors declare that the research was conducted in the absence of any commercial or financial relationships that could be construed as a potential conflict of interest.

## Publisher's Note

All claims expressed in this article are solely those of the authors and do not necessarily represent those of their affiliated organizations, or those of the publisher, the editors and the reviewers. Any product that may be evaluated in this article, or claim that may be made by its manufacturer, is not guaranteed or endorsed by the publisher.
